# Cell wall integrity signaling and innate immunity in plants

**DOI:** 10.3389/fpls.2012.00280

**Published:** 2012-12-11

**Authors:** Thomas S. Nühse

**Affiliations:** Faculty of Life Sciences, University of ManchesterManchester, UK

**Keywords:** cell wall, cell wall integrity, immunity, signaling, danger-associated molecular pattern, receptor-like kinase

## Abstract

All plant pathogens and parasites have had to develop strategies to overcome cell walls in order to access the host’s cytoplasm. As a mechanically strong, multi-layered composite exoskeleton, the cell wall not only enables plants to grow tall but also protects them from such attacks. Many plant pathogens employ an arsenal of cell wall degrading enzymes, and it has long been thought that the detection of breaches in wall integrity contributes to the induction of defense. Cell wall fragments are danger-associated molecular patterns or DAMPs that can trigger defense signaling pathways comparable to microbial signals, but the picture is likely to be more complicated. A wide range of defects in cell wall biosynthesis leads to enhanced pathogen resistance. We are beginning to understand the essential role of cell wall integrity surveillance for plant growth, and the connection of processes like cell expansion, plasma membrane–cell wall contact and secondary wall biosynthesis with plant immunity is emerging.

The cell wall represents a unique challenge for pathogens specializing in plants. Manipulation of the host, delivery of effectors and suppression of defense responses requires intimate contact between parasite and host. Cell wall polysaccharides – cellulose, pectin, and hemicelluloses such as xyloglucan and arabinoxylan – are potentially a major source of carbon but are difficult to access. Depending on their lifestyle, some pathogens extensively degrade cell walls, such as the macerating necrotrophs *Erwinia* or *Botrytis*; others puncture it with surgical precision, such as biotrophic fungal and oomycete pathogens during the formation of appressoria. Dissolving and rearranging cell walls is also part of the large-scale host manipulation undertaken by plant parasitic nematodes establishing feeding sites (Gheysen and Mitchum, 2011). It seems obvious that such breaches of cell wall integrity (CWI) should alert the host plant to the presence of invaders. Among the potential warning signs are changes in mechanical properties, interference with cell wall proteins or polysaccharides by the binding of effectors, and release of oligosaccharide fragments with DAMP (danger-associated molecular pattern) activity. However, the relative contribution made by each of these signals toward mounting efficient defense responses is still unclear. In the last few years, the concept of CWI signaling in plants has matured. While the close link of this pathway with innate immunity has been instrumental in its discovery, maintenance of mechanical CWI is also a necessary part of controlled cell expansion in healthy plants.

## THE NEED FOR CELL WALL MAINTENANCE

Plant cell walls and the pressurized cells within them represent an economic solution for growing a multicellular organism: Without a proportional metabolic investment in cytoplasmic material, cells can grow simply by accumulating water and solutes in the vacuole and then driving expansion of the wall via turgor pressure. Cell walls need to remain strong throughout this expansion and yet yield in a controlled way (Cosgrove, 2005). In some extreme cases, such as the expanding primary root tip or the hypocotyls of etiolating seedlings, this expansion increases the cell surface by an order of magnitude within hours (Beemster and Baskin, 1998). Many other developmental programmes require irreversible cell wall weakening or dissolution, including the emergence of lateral roots and of the radicle from the seed coat; formation of vasculature, stomata, and aerenchyma; abscission, organ separation, and fertilization. The controlled yielding of cell walls during expansion requires a way of feeding back information about wall stability to the cytoplasm so that growth rates can be adjusted if necessary. Root cell elongation, for example, is known to be influenced by a wide range of environmental factors (De Cnodder et al., 2006), confirming that the developmental programme integrates external information rather than unfolding by default. The nature of this surveillance system and the postulated CWI sensors is actively debated (Ringli, 2010; Seifert and Blaukopf, 2010). It is already clear, however, that surveillance of plant cell wall structure and innate immunity are closely linked.

## THE CELL WALL AS A BARRIER FOR PATHOGENS

Cell wall degrading enzymes are a major part of the weaponry used by necrotrophic and, to a lesser extent, biotrophic pathogens (Walton, 1994). The tightly packed crystalline arrangement of microfibrils makes cellulose an unattractive target for attack. In contrast, pectin and xylan, major components of type I cell walls in most dicots and type II walls in most grain crops respectively, are easier to access and break down. Enzymes degrading pectin (polygalacturonases, pectate lyases, and pectin methyl esterases) and xylan (endo-xylanases) are key virulence factors for pathogens. In turn, plants counter these attacks with an array of inhibitor proteins (Juge, 2006). Interestingly, the function of polygalacturonase inhibitor proteins seems not primarily to block pectin degradation completely but to shift the breakdown process toward generating larger fragments that are DAMP active (Federici et al., 2006).

Natural infection routes chosen by plant pathogens often reflect how the cell wall acts as a barrier. For example, soil borne fungi typically first colonize a root at the tip but can only invade the root in the elongation zone where walls are temporarily weakened and thinned (Gunawardena and Hawes, 2002). Fruit ripening is another example for easier pathogen entry in areas of developmentally regulated cell wall weakening. Polygalacturonases and pectate lyases contribute substantially to the softening of fruit. Suppression of these enzymes delays fruit softening and at the same time confers enhanced resistance to pathogens like *Botrytis* (summarized in Cantu et al., 2008). Similarly, promoting cell wall stiffness by overexpressing extensin in *Arabidopsis* enhanced resistance to *Pseudomonas syringae* (Wei and Shirsat, 2006). In other cases, changes in cell wall composition increase susceptibility to a pathogen in ways that are more difficult to explain. The receptor-like kinase (RLK) ERECTA is a major determinant of resistance to the necrotrophic pathogens *Ralstonia solanacearum* and *Plectosphaerella cucumerina*. The *erecta *mutant has increased cellulose and uronic acid contents in the cell wall (Godiard et al., 2003; Llorente et al., 2005; Sanchez-Rodriguez et al., 2009). Similarly, mutants in the alpha and beta subunits of heterotrimeric G-proteins are more susceptible to *P. cucumerina* and have a subtly altered cell wall structure including less xylose (Llorente et al., 2005; Delgado-Cerezo et al., 2011). It is unclear how cell wall composition is controlled by these signaling proteins, but the positive correlation of increased uronic acid and decreased xylose with susceptibility to *P. cucumerina *has been confirmed in additional mutants (Sanchez-Rodriguez et al., 2009; Delgado-Cerezo et al., 2011).

## DISEASE RESISTANCE TRIGGERED BY CELL WALL DEFECTS

There are many other cases of cell wall alterations or defects that – perhaps counterintuitively – enhance pathogen resistance. Some of these are subtle shifts in polysaccharide composition that may reduce the suitability of the host’s wall for pathogen attachment or ingress, i.e., may be susceptibility factors. Several of the *powdery mildew resistant* (*pmr*) mutants may fall into this category (Vogel et al., 2002, 2004). Both *pmr5*, mapped to one member of a large plant-specific gene family related to TRICHOME BIREFRINGENT (Bischoff et al., 2010) and *pmr6*, a pectate lyase mutant, have increased levels of unesterified pectin and activate resistance via an unknown pathway that is independent of the well-studied salicylic acid (SA), ethylene (ET), or jasmonic acid (JA)-responsive paths. The *pmr5* and *pmr6* mutants only have slightly enhanced constitutive defense responses relative to the wild-type. In contrast, resistance to *Erysiphe cichoracearum *in *pmr4*, a callose synthase (Nishimura et al., 2003), and resistance to *Hyaloperonospora parasitica *in *cie1/ mur3*, a putative xyloglucan galactosyltransferase (Tedman-Jones et al., 2008), is based on constitutive activation of SA-dependent defense responses. The clearest indication of a causal link between cell wall defects and activation of defense responses came from the discovery of a series of mutants in cellulose synthase proteins that confer enhanced resistance to either biotrophic or necrotrophic pathogens. Two allelic mutations in the primary wall cellulose synthase gene CesA3 were identified in genetic screens for ectopic lignin deposition in the root (*eli1*) and on the basis of constitutive expression of the JA-induced gene vsp1 (*cev1*), respectively (Cano-Delgado et al., 2000; Ellis et al., 2002). Resistance to powdery mildew is considerably higher in *cev1 *than the wild-type and requires JA and ET (Ellis and Turner, 2001). In contrast, defects in cellulose synthase proteins required for secondary cell wall formation (CesA4/IRX5, CesA7/IRX3, and CesA8/IRX1) confer enhanced resistance to the necrotrophic pathogens *P. cucumerina* and *R. solanacearum *in a pathway requiring ABA signaling but neither SA nor JA/ET (Hernandez-Blanco et al., 2007). Several other mutants in cell wall-related genes have since been discovered that also show variable degrees of resistance to pathogens or constitutive expression of defense-related genes (Ko et al., 2006; Vega-Sanchez et al., 2012). Drugs that interfere with cellulose biosynthesis, such as isoxaben and thaxtomin, phenocopy this response (Bischoff et al., 2009; Hamann et al., 2009). These discoveries sparked the idea of cell wall feedback signaling: a dedicated signaling pathway that monitors the physical integrity and functioning of the cell wall and if necessary activates repair responses.

## THE CELL WALL INTEGRITY PATHWAY IN PLANTS

Loss of CWI, triggered by genetic defects in polysaccharide biosynthesis or by drugs, reduces cell elongation in etiolated hypocotyls and root tips (Hauser et al., 1995; Desnos et al., 1996; Desprez et al., 2002). If this response is based on a signaling process rather than physical inability to elongate, it should be possible to uncouple cell wall damage from its effect on expansion by blocking the signaling pathway. Experimental evidence shows that this is indeed the case (Refregier et al., 2004; Hematy et al., 2007; Tsang et al., 2011; Wolf et al., 2012). Mutation of the receptor-like kinase THESEUS attenuates the cell expansion defect of *procuste*, a mutant in a primary wall cellulose synthase (Fagard et al., 2000; Hematy et al., 2007). Several other (though not all tested) cell wall-deficient mutants are also rescued in a *the1* mutant background. In seedlings treated with isoxaben, the production of reactive oxygen species and lignin deposition is partially dependent on THE1 (Denness et al., 2011). THESEUS is only one of a whole range of potential cell wall sensors. Many others have been suggested based largely on the predicted (and in a few cases demonstrated) ability to bind cell wall components and transmit a signal to the cytoplasm. The rationale follows the well-characterized CWI pathway in yeast (Levin, 2011). Here, plasma membrane (PM) proteins including Wsc1 and Mid2 extend stiff hyper-glycosylated “antennae” into the wall and transmit signals with their short cytoplasmic domains. In the absence of obvious plant homologs of these sensors, the most attractive candidates are RLKs. In addition to THESEUS, several other members of the CrRLK1L (*Catharanthus roseus *RLK1-like) family of RLKs with an extracellular malectin-like domain have well-documented cell wall-related functions (for review, see Boisson-Dernier et al., 2011): FERONIA and ANXUR are required in the female and male gametophyte, respectively, for successful fertilization. Pollen tube guidance by the synergid cells and sperm release fail in *feronia* while pollen tubes burst prematurely in *anxur1/2* double mutants. FER, THE, and the related HERKULES1 and 2 are brassinosteroid-inducible and have partially redundant roles in cell expansion throughout the plant (Guo et al., 2009).

Intriguingly, *feronia* mutants are more resistant to powdery mildew infection (Kessler et al., 2010), based perhaps on the mechanistic similarities between fertilization and fungal invasion. Both involve polarization of membrane proteins toward the pollen tube and fungal hyphae/appressoria, respectively. With the exception of the wall-associated kinases (WAKs, see below) and FER, it is not known whether any other candidate cell wall sensors have a role in immunity, such as the leucine-rich repeat (LRR-) RLKs, FEI1 and FEI2. The *fei1fei2 *mutant has a characteristic conditional root expansion phenotype and impaired cellulose biosynthesis (Xu et al., 2008) that points to a role in cell wall homoeostasis for these RLKs.

PM–cell wall contacts have a key role in plant resistance to fungal penetration (Mellersh and Heath, 2001). These contacts, visible as Hechtian strands during plasmolysis, can be dissociated by addition of RGD (Arg-Gly-Asp) peptides like in metazoans (Canut et al., 1998). The existence of high-affinity binding sites for the RGD sequence in plants has long been puzzling because plants appeared to have neither fibronectin-like (RGD ligand) nor integrin-like (RGD receptor) proteins. RGD sequence motives are present on several oomycete effector proteins such as IPI-O of *Phytophthora *and are essential for attachment to the host (Senchou et al., 2004). Two recent developments have shed light on the connection: The *Arabidopsis* lectin-like receptor kinase LecRK-I.9 has been identified as a receptor for RGD peptides. Null mutants have reduced membrane–wall contacts, increased susceptibility to *Phytophthora brassicae* and almost no callose deposition in response to effector-disabled *Pseudomonas syringae* or bacterial flagellin. All these effects are phenocopied by overexpression of the RGD-motif effector, IPI-O (Bouwmeester et al., 2011). In a different study, Knepper et al. (2011) showed that NDR1, a PM protein required for several race-specific resistance pathways, also mediated PM–cell wall adhesion depending on its own Asn-Gly-Asp (NGD) motif. It is tempting to speculate that LecRK-I.9 binds to the NGD motif on NDR1, although that leaves the question open how association of two PM proteins establishes contact with the cell wall.

NDR1 and RLKs are not the only candidates for signaling proteins with a cell wall–cytoplasm bridging function. Class I formin homology proteins are membrane-anchored proteins with the ability to organize the actin cytoskeleton. The proline-rich extracellular domain of AtFH1 has been shown to bind to the cell wall (Martiniere et al., 2011). AtFH1 and the closely related AtFH6 are induced in the early stages of giant cell formation triggered by the plant–parasitic root knot nematode, *Meloidogyne incognita *(Favery et al., 2004). These proteins are ideal candidates for transmitting mechanical stress across the PM. The central role of the cytoskeleton in cell wall biosynthesis (Paredez et al., 2006), plant cell morphogenesis (Szymanski and Cosgrove, 2009), and innate immunity (Hardham et al., 2007) is well-recognized. Despite this connection, cytoskeletal functions in plant CWI signaling have not been studied extensively. In the yeast CWI pathway, the formins Bni1 and Bnr1 are key effectors of actin rearrangement and bind directly to the central regulator GTP-Rho1 (Levin, 2011).

The exact nature of the signal that communicates deficient cell walls is a matter of intense debate and may not be (exclusively) based on a direct polysaccharide sensor. Because of the turgor pressure, weakening cell walls will lead to unplanned protoplast expansion and PM stretch. Some responses triggered by inhibition of cellulose biosynthesis do indeed depend on the osmosensors Cre1 and Mca1 (Wormit et al., 2012) while others do not. Oligosaccharide fragments released from wall polysaccharides may represent another damage or danger signal. Specifically in the context of pathogen attack, some of the cell wall degrading enzymes released by microbial parasites have *endo*-activity and will set free such fragments. Short oligogalacturonides (DP 6-16) have long been known to induce rapid and strong defense responses (Doares et al., 1995). Wall-associated protein kinases (WAKs) have now been identified as likely receptors (Kohorn et al., 2009; Brutus et al., 2010). The *WAK*s, a family of RLKs with extracellular fibronectin-type repeats, also play a role in cell wall maintenance in normal plant development (Wagner and Kohorn, 2001; Kohorn et al., 2006), and a differential affinity for low- and high-molecular weight pectins may allow for a dual role in pathogen detection versus cell wall maintenance during growth (Kohorn and Kohorn, 2012). A WAK-like kinase (WAKL22) is a major determinant of resistance to *Fusarium oxysporum* in *Arabidopsis* (Diener and Ausubel, 2005). No specific detection systems for other types of endogenous wall fragments have been identified. Cellodextrins (i.e., β-1,4-linked glucose oligomers conceivably derived from cellulose) and β-1,3-glucan fragments trigger defense responses in grapevine cell cultures (Aziz et al., 2007). However, like oligogalacturonides they only do so in much higher concentrations than comparable “non-self” oligosaccharides such as chitin (Felix et al., 1993). It is likely that sensors for cross-linked cell wall polysaccharides as well as sensors for fragments derived from them play a part in plant CWI signaling, but relative contributions are still completely open.

## THE ROLE OF PROTEOMICS IN DECIPHERING THE CWI PATHWAY

Analyzing the subcellular processes during pathogen invasion is difficult with proteomic tools – processes like cell polarization only occur in the attacked cells, and sampling only these is extremely challenging. However, just as the response to bacterial flagellin has been a useful model system for studying defense responses using proteomics and phosphoproteomics (Nühse et al., 2007), low molecular weight compounds can be used to induce cell wall defects (Hamann et al., 2009; Tsang et al., 2011) that phenocopy those observed in cell wall biosynthetic mutants (see above). Signaling proteins identified as differentially phosphorylated in such a setup are very likely to have roles both in normal plant growth and cell wall-based defense against pathogens.

Intriguing links between normal development, cell wall homoeostasis and innate immunity have emerged with the discovery of novel roles for ERECTA and NDR1 (Sanchez-Rodriguez et al., 2009; Knepper et al., 2011). The identification of binding partners (Roux et al., 2011) of these and other proteins, especially putative cell wall sensors, will be a challenge–like mature WAKs, wall-associated proteins may have “the biochemistry of a rock” (B. Kohorn, unpublished). We need to take on this challenge to advance our understanding of signaling networks connecting immunity and CWI.

## Conflict of Interest Statement

The author declares that the research was conducted in the absence of any commercial or financial relationships that could be construed as a potential conflict of interest.
